# A novel computed tomography angiography technique: guided preoperative localization and design of anterolateral thigh perforator flap

**DOI:** 10.1186/s13244-022-01318-0

**Published:** 2022-12-13

**Authors:** Xin-xin Yu, Shi-feng Yang, Cong-shan Ji, Shen-qiang Qiu, Yao-dong Qi, Xi-ming Wang

**Affiliations:** 1 Department of Radiology, Shandong Provincial Hospital, Shandong University, No. 324, Jingwu Road, Jinan, 250021 Shandong China; 2 Department of Radiology, Shandong Provincial Hospital Affiliated to Shandong First Medical University, No. 324, Jingwu Road, Jinan, 250021 Shandong China; 3 Department of Hand and Foot Surgery, Shandong Provincial Hospital Affiliated to Shandong First Medical University, No. 324, Jingwu Road, Jinan, 250021 Shandong China

**Keywords:** Computed tomography angiography, Imaging techniques, Thigh, Perforator flap, Reconstructive surgical procedures

## Abstract

**Background:**

Anterolateral thigh perforator (ALTP) flap is considered a versatile flap for soft tissue reconstruction. Computed tomography angiography (CTA) is used for mapping perforator in abdominal-based reconstruction; however, it is less commonly used in ALTP due to its poor imaging efficacy. In this study, we introduced a novel CTA technique for preoperative localization and design of ALTP flap and evaluated its value in directing surgical reconstruction.

**Results:**

Thirty-five patients with soft tissue defects were consecutively enrolled. Modified CTA procedures, such as sharp convolution kernel, ADMIRE iterative reconstruction, 80 kV tube voltage, high flow contrast agent and cinematic rendering image reconstruction, were used to map ALTPs. A total of 287 perforators (including 884 sub-branches) were determined, with a mean of 5 perforators per thigh (range 2–11). The ALTPs were mainly concentrated in the “hot zone” (42%, 121/287) or the distal zone (41%, 118/287). Most perforators originated from the descending branch of the lateral circumflex femoral artery (76%, 219/287). Three perforator types, namely musculocutaneous (62%, 177/287), septocutaneous (33%, 96/287), and mixed pattern (5%, 14/287), were identified. The median pedicle length measured by two methods was 4.1 cm (range 0.7–20.3 cm) and 17.0 cm (range 4.7–33.9 cm), respectively, and the median diameter of the skin flap nourished by one perforator was 3.4 cm (IQR 2.1–5.7 cm). Twenty-eight ALTP flaps were obtained with the guidance of CTA, and 26 flaps survived after follow-up.

**Conclusions:**

The proposed CTA mapping technique is a useful tool for preoperative localization and design of ALTP flap.

## Key points

- Sharp convolution kernel combined with ADMIRE iterative reconstruction improves the imaging efficacy.
- 80 kV tube voltage with high flow contrast agent ensures the perforator enhancement.
- Cinematic rendering reconstruction model facilitates the visualization of perforator anatomic variations.
- Preoperative CTA mapping contributes to the localization and design of the ALTP flap.

## Background

The anterolateral thigh perforator (ALTP) flap is a versatile flap and one of the most popular procedures for reconstructing three-dimensional defects in the extremities. Its major advantages are pliable skin paddle, long vascular pedicle, large soft tissue volume, and low donor site morbidity [1–4]. The position of ALTPs is related to the midpoint of a line from the anterior superior iliac spine to the superior lateral boundary of the patella (A–P line) [5, 6]. However, the anatomic variation and distribution pattern of perforators in this area varies, which makes it difficult to precisely predict the location of perforators for flap harvest [7–9].

So far, several imaging modalities have been proposed to map the anatomic distribution of ALTPs, such as computed tomography angiography (CTA), magnetic resonance angiography, and Doppler ultrasound [10–12]. Compared with magnetic resonance angiography and Doppler ultrasound, CTA has a higher temporal and spatial resolution and is more accurate in showing perforator vessels, especially small ones. Furthermore, three-dimensional image post-processing facilitates the visualization of anatomical structures in space, including the origin, course, and emerging location of perforators, which is helpful for surgeons in selecting the optimal perforator flap and surgical approach. However, although abundant literature supports the routine use of preoperative CTA to identify the dominant perforator flap, traditional CTA procedures have poor imaging efficacy for ALTP [13, 14], especially for the tiny perforator at the distal branch.

In 2016, Dappa et al. [15] first proposed using a cinematic rendering (CR), a novel three-dimensional technique for post-processing based on computed tomography image data. The main innovation in CR images is a more photorealistic representation of high density and high contrast structures such as bones and contrast-enhanced vessels. In 2019, Elshafei et al. [16] evaluated the value of CR for the comprehension of the surgical anatomy in 40 German patients treated or followed up for hepatopancreatobiliary tumors and found that CR allows a faster and correct comprehension compared with conventional computed tomography images, and the results were independent of the level of surgeon experience. Yet, so far, no studies have reported using CR to characterize of ALTP. Also, only a few studies have used CTA data to mark the location of perforators.

In this study, we introduced a novel CTA technique for preoperative localization and design of ALTP flap and evaluated its value in directing surgical reconstruction.

## Materials and methods

### Patients

Patients with soft tissue defects caused by trauma or tumor resection who underwent CTA between June 2020 and August 2021 were consecutively enrolled in this retrospective study. The exclusion criteria were: (1) patients with a history of abnormal renal function or allergic reaction to an iodinated contrast agent; (2) patients with acute vascular injury; (3) presence of clinically significant pathology in bilateral thigh (including trauma, tumor, or infection); and (4) insufficient imaging or presence of motion/metal artifacts which could affect image analysis.

The study was approved by the institutional review board and was conducted in accordance with the Declaration of Helsinki. The informed consent was waived.

### Image acquisition

A modified CTA of the lower extremities from the pelvis to below the knee was performed using the third-generation dual-source computed tomography scanner (Somatom Force, Siemens Healthcare, Forchheim, Germany). The reticular position lines were drawn on patient’s thigh prior to examination. Immediately before CTA acquisition, all subjects received sublingual nitroglycerin (0.1 mg per dose; Nitroglycerin Inhaler, Jingwei Pharmacy Co, Ltd, Jinan, China). The details of the CTA acquisition are shown in Table 1.

**Table 1:** The details of the CTA acquisition

Parameter	
Nitroglycerin	Sublingual (0.1 mg per dose)
Detector collimation (mm)	96 × 2 × 0.6
Gantry rotation time (s/r)	0.5
Tube voltage (kV)	80
Tube current modulation	Automated tube current (CARE Dose 4D)
Matrix	512 × 512
Pitch	0.35
Vision (mm^2^)	≤ 170
Convolution reconstruction kernel	Br54
Iterative reconstruction	ADMIRE (strength 5)
Slice thickness (mm)	0.75
Slice increment (mm)	0.3
Bolus-tracking monitoring section	The middle segment of the descending branch with a “manual” trigger mode
Injection speed (ml/s)	6–7.5
Contrast agent volume (ml)	90–112.5

Iohexol (350 mg I/ml, Beilu Pharmaceutical, China) was injected via the median cubital vein at a flow rate of 6–7.5 ml/s for 15 s. Saline solution was immediately given at the same flow rate for 8 s. A “manual” bolus-tracking technique was used, and the monitoring section was set at the middle segment of the descending branch of the lateral circumflex femoral artery (LCFA). The region of interest (ROI) was placed outside the body. Image acquisition was manually triggered with a delay of 3 s after the descending branch of LCFA appeared.

The dose-length product was obtained from the scan protocol. The effective dose was calculated according to the product of the dose-length product and a conversion factor “k” [17].

### Image post-processing

All images were transferred to an external workstation (Syngo.Via, Siemens Healthcare, Forchheim, Germany) for maximum intensity projection, multiplanar reformation, and CR. A CR reconstruction model was based on voxel data for segmentation. First, enhanced veins in the skin and superficial fascia were manually removed, and the LCFA and its perforators were displayed inside the deep fascia. Second, the emerging location of the perforator was determined in the axial plain and marked at the deep fascial level. Then, three types of volume reproduction images were used to display different organizational structures by adjusting the template. Ultimately, the emerging location of perforators was marked by projection onto the skin.

### Parameter measurement and assessment

The number, position, origin, course, caliber, and pedicle length of the perforator in reconstructed and axial images were determined and evaluated by two senior radiologists (S.F.Y. and C.S.J., each with 6 years of experience in CTA) in a consensus reading. As for the location distribution of perforators, the “hot zone” was defined as the area covered by 5 cm radius from the midpoint of the A–P line. The proximal and distal zones were defined as the proximal and distal areas of the “hot zone,” respectively [10]. The course of perforators was divided into septocutaneous, musculocutaneous, or a mixed pattern, as previously described [10, 18]. The caliber was defined as small (diameter ≤ 0.5 mm), medium (diameter 0.5–1 mm), and large (diameter ≥ 1 mm). Two measurement methods were used for pedicle length, namely the minimum and maximum lengths that can be dissected [9, 13, 14, 19]. The minimum length was the distance of the emerging point to the end of the perforator, while the maximum length was defined as the distance from the emerging point to the origin of main upstream vessel. Meanwhile, the diameter of the skin flap nourished by each perforator was measured.

### Image quality assessment

Subjective image quality was assessed by two experienced observers (S.F.Y. and C.S.J., each with 6 years of experience in CTA) independently using a five-point scale [20]: 1 = poor, 2 = fair, 3 = moderate, 4 = good, and 5 = excellent. Meanwhile, objective image quality was evaluated on the axial images. One observer (C.S.J) placed the ROIs on the middle segment of the descending branch of the LCFA and muscle of the same plane respectively, and the image noise was defined as the SD of attenuation in a round ROI (area = 1 cm^2^). Measurement was performed three times for each target, and the mean attenuation and image noise were obtained. The contrast-to-noise ratio was calculated as the difference between the average attenuation of the target vessel and muscle divided by the image noise [21–23].

### Perforator flap design and harvesting

The surgical planning for perforator flap harvesting was based on the preoperative localization of CTA. The skin paddle was designed with the dominant perforators at the center of the flap. The flap dissection was performed when the dominant perforator and its origin and pathway were determined by surface projection,

### Statistical analysis

Statistical analyses were performed using the SPSS software package (version 25.0, SPSS, Chicago, IL, USA). Kolmogorov–Smirnov analysis was used in assessing the normality distribution of data. All continuous variables were expressed as mean ± standard deviation or median and interquartile range (IQR), whereas categorical variables were expressed as a number with a percentage. In addition, intraclass correlation coefficient was used for calculating interobserver agreement for image quality. A *p* value < 0.05 indicated statistical significance.

## Results

### Patient demographics

**Fig. 1:**
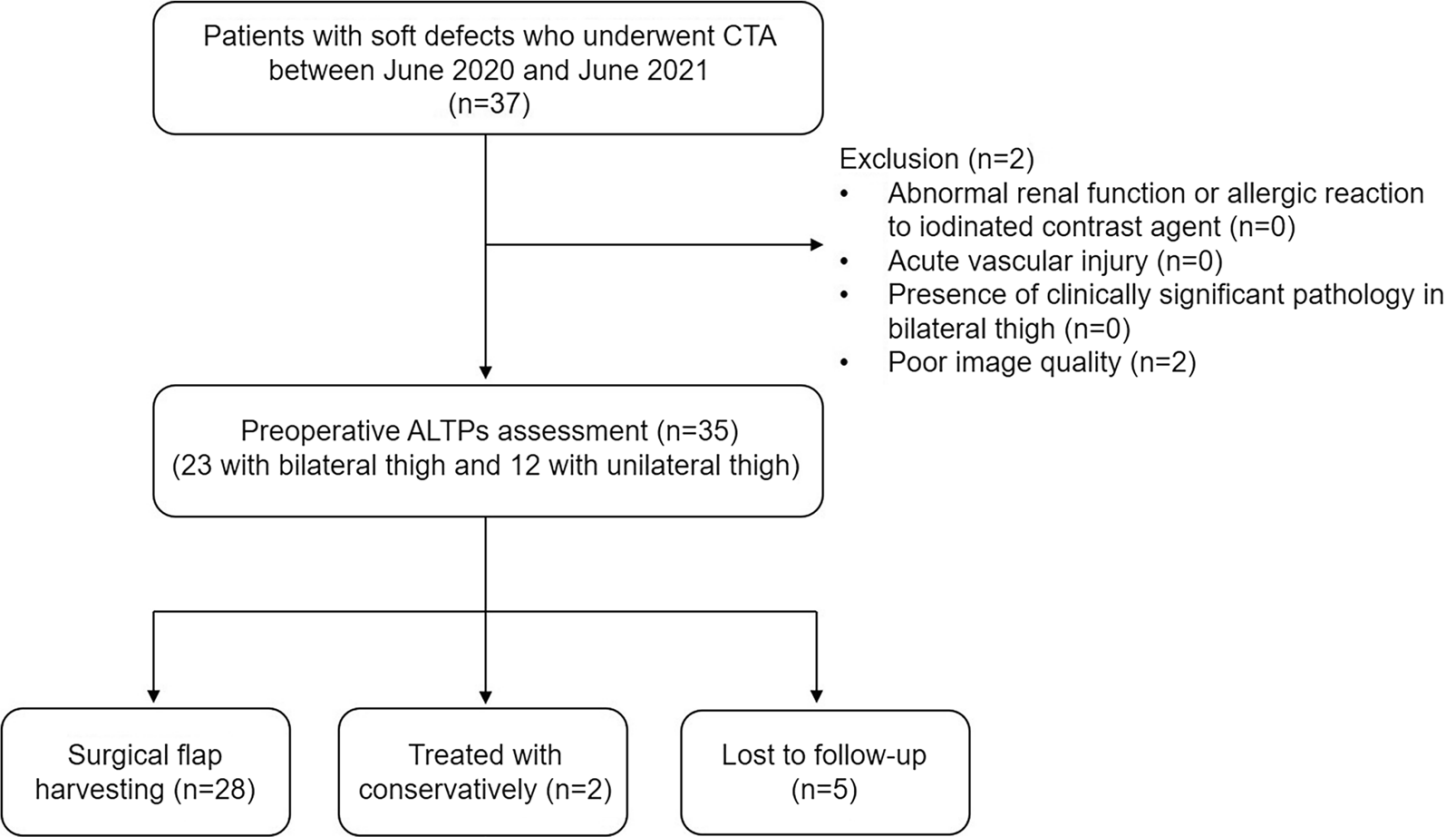
Study flow diagram

A total of 37 patients with soft tissue defects caused by trauma or tumor resection who underwent CTA were enrolled in this study. Two patients were excluded due to the presence of metal artifacts in the images. Finally, 35 patients (median age, 48 years; IQR 32–54 years; 19 men) were included in the data analysis, 23 patients with bilateral thigh, and 12 with the unilateral thigh. The flow diagram for this study is shown in Fig. 1. The mean dose-length product for patients was 519.5 ± 96.5 mGy.cm corresponding to a mean effective dose of 2.7 ± 0.6 mSv (Table 2).

**Table 2:** Patient demographics

Parameter	Value (*n* = 35)
Patient characteristics	
Median age (y)*	48 (32–54)
Men^†^	19 (54%)
Radiation	
DLP (mGy.cm)	519.5 ± 96.5
ED (mSv)	2.7 ± 0.6
Image quality	
Subjective score*	4 (4–4.5)
CNR	25.3 ± 10.6

Unless otherwise specified, data are mean ± standard deviation. * Data are median value, with interquartile range in parentheses. ^†^ Data are presented as a number (%). CNR, contrast-to-noise ratio; DLP, dose-length product; and ED, effective dose

### Preoperative CTA information of perforators

#### Number and location distribution

**Fig. 2:**
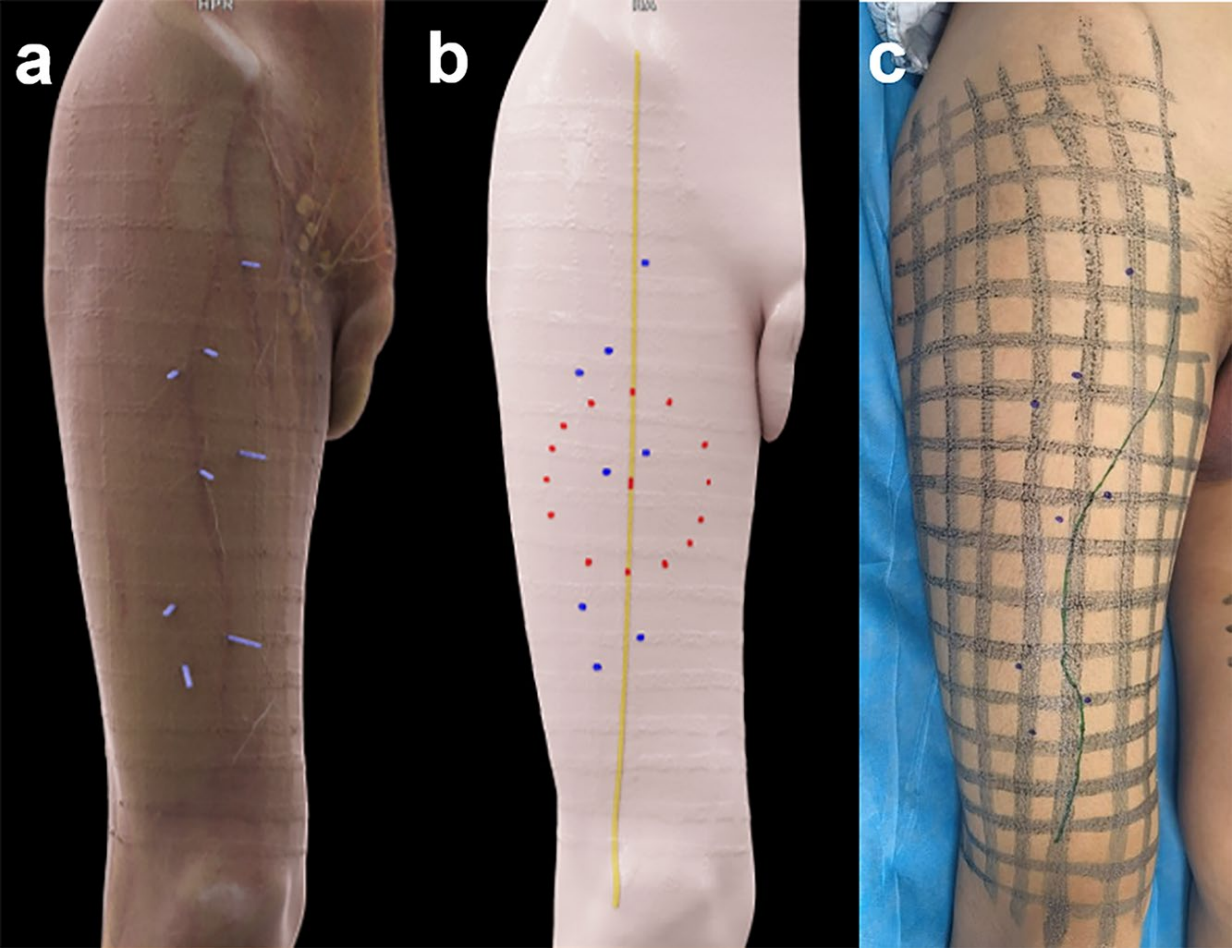
CTA mapping for ALTPs. **a** Vertical projection of perforators by cinematic rendering reconstruction. **b** A diagram depicting the position distribution of perforators. The “hot zone” (red points) was an area of 5-cm radius from the midpoint between the anterior superior iliac spine and the superior lateral boundary of the patella (yellow line); the location of perforators (blue points) was marked. **c** The course of the descending branch of the lateral femoral circumflex artery and the location of perforators drawn on the skin

Overall, 287 ATLPs of 35 patients were identified based on CTA results, with a mean of 5 perforators per thigh (range 2–11) (Table 3). Besides, 884 sub-branches were found to nourish the skin flap, with a mean of 3 sub-branches per perforator. One hundred and twenty-one perforators were detected in the “hot zone,” 48 perforators in the proximal zone, and 118 perforators in the distal zone. Two patients had no perforator in the “hot zone.” The CTA mapping for ALTPs is shown in Fig. 2.

**Table 3:** Quantitative parameter of anterolateral thigh perforators

Parameter	Value (*n* = 287)
Position	
Proximal zone	48 (17%)
“Hot zone”	121 (42%)
Distal zone	118 (41%)
Origin	
Descending branch of LCFA	219 (76%)
Oblique branch of LCFA	23 (8%)
Ascending branch of LCFA	17 (6%)
Transverse branch of LCFA	11 (4%)
CFA	4 (1%)
DFA	11 (4%)
PA	2 (1%)
Course	
Musculocutaneous	177 (62%)
Septocutaneous	96 (33%)
Mixed pattern	14 (5%)
Caliber (mm)	
≤ 0.5	89 (31%)
0.5–1	131 (46%)
≥ 1	67 (23%)
Pedicle length (cm)*	
Minimum length	4.1 (3.0–6.4)
Maximum length	17.0 (12.0–22.6)
Diameter of skin flap nourished by one perforator (cm)*	3.4 (2.1–5.7)

Unless otherwise specified, data are presented as numbers (%). * Data are median values, with interquartile ranges in parentheses. LCFA, lateral circumflex femoral artery; CFA, common femoral artery; DFA, deep femoral artery; and PA, popliteal artery

#### Origin and course

**Fig. 3:**
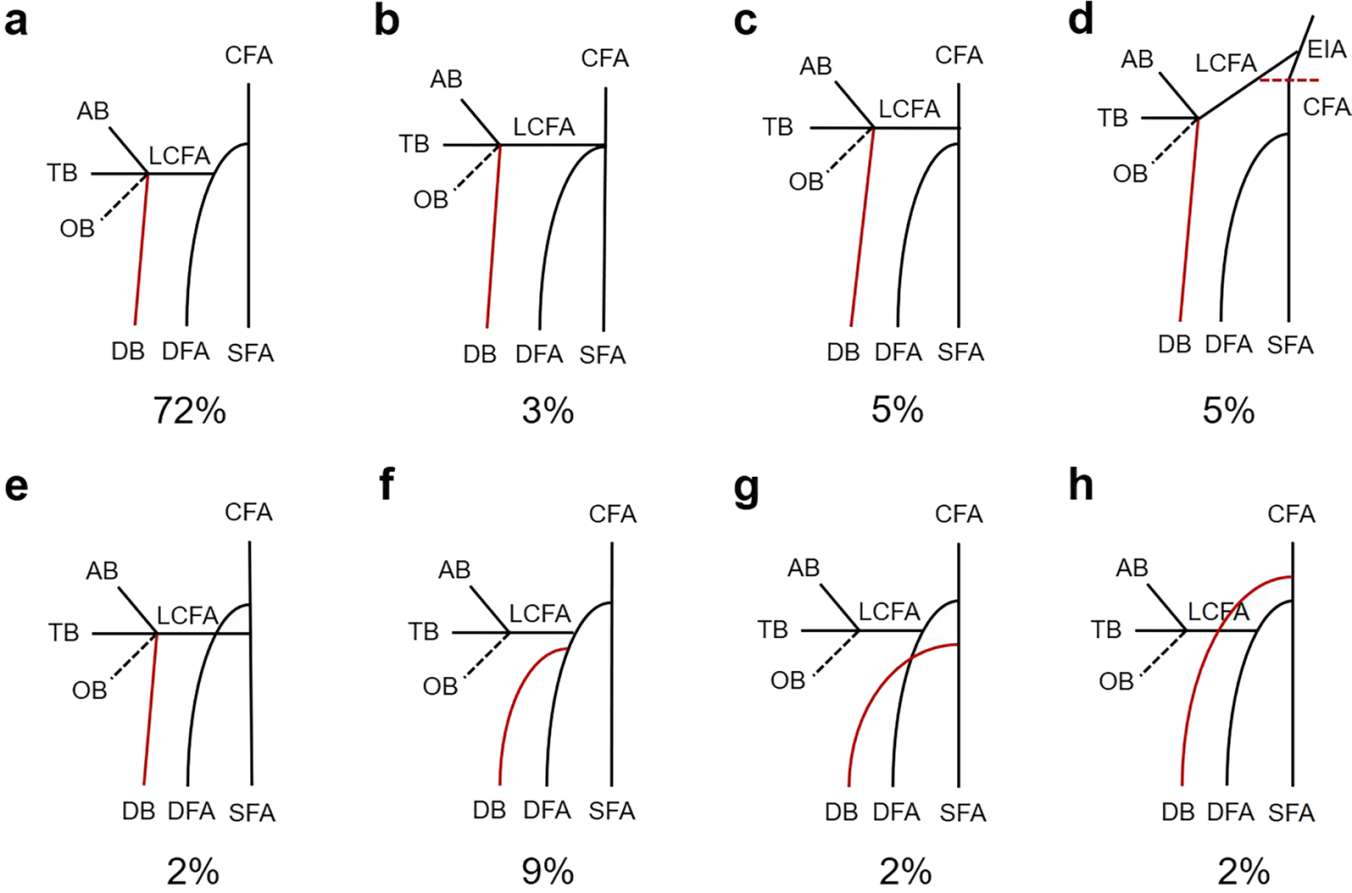
Different origin variation types and percentages of the descending branch. **a–e** The descending branch originated from the lateral circumflex femoral artery, while the lateral circumflex femoral artery originated from different superior vessels, such as the deep femoral artery, the common femoral artery, the external iliac artery, and the superficial femoral artery. **f** The descending branch originated from the deep femoral artery. **g** The descending branch originated from the superficial femoral artery. **h** The descending branch originated from the common femoral artery. Notes: Data are presented as %. EIA, external iliac artery; CFA, common femoral artery; SFA, superficial femoral artery; DFA, deep femoral artery; LCFA, lateral circumflex femoral artery; AB, ascending branch; TB, transverse branch; OB, oblique branch; and DB, descending branch

In this series, most ALTPs originated from the descending branch of the LCFA (76%, 219/287). Eight percent (23/287) of the perforators originated from the oblique branch, six percent (17/287) from the ascending branch, four percent (11/287) from the transverse branch, and six percent (17/287) directly from the other main vessels. The descending branch mainly originated from the LCFA (88%, 51/58). Importantly, the anatomic variants were observed in seven cases with the descending branch directly from the common femoral artery (CFA), superficial femoral artery, or deep femoral artery. However, the majority of LCFA (83%, 48/58) originated directly from the deep femoral artery, followed by the CFA (12%, 7/58) and the external iliac artery (5%, 3/58). Regarding the course of perforators, 177 musculocutaneous perforators, 96 septocutaneous perforators, and 14 mixed patterns were found. Different origin variation types of the descending branch are shown in Fig. 3.

#### Caliber and pedicle length

**Fig. 4:**
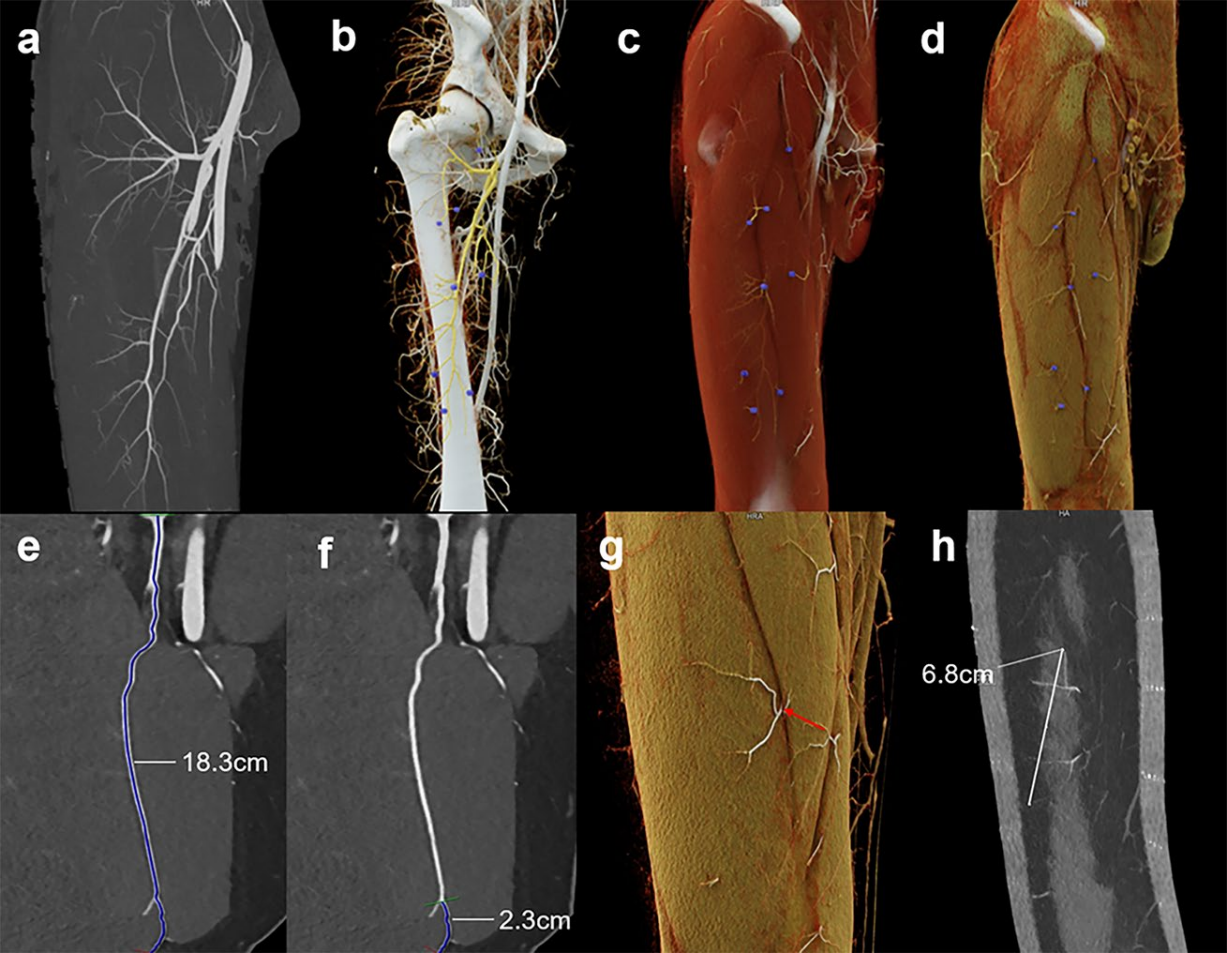
Description of anatomical characteristics and parameter measurement of ALTPs. **a–d** Maximum intensity projection image (**a**) and cinematic rendering images (**b–d**) represented by a three-tier organization structure (from deep blood vessel to subcutaneous tissue). A solitary origin of the descending branch directly from the deep femoral artery was identified. A total of eight perforators (five septocutaneous perforators and three musculocutaneous perforators) were found; two perforators originated from the ascending branch and the remaining six perforators from the descending branch. The emerging point of perforators through deep fascia was marked in cinematic rendering images. **e–h** Measurement of maximum length (18.3 cm) of perforator from the origin of descending branch to the emerging point at deep fascia (**e**) and minimum length (2.3 cm) of perforator (**f**). The emerging point of a perforator containing two main sub-branches in distal was marked by a red arrow (**g**), and the diameter of a skin flap nourished by this perforator was 6.8 cm (**h**)

In terms of the caliber, there were 198 perforators with a diameter of ˃ 0.5 mm and 89 perforators with a diameter of ≤ 0.5 mm. Twenty-nine patients had more than one perforator with a caliber of ˃0.5 mm in the “hot zone”, and 4 patients only had small perforators in this area. The minimum lengths of perforators ranged from 0.7 cm to 20.3 cm (median, 4.1 cm; IQR 3.0–6.4 cm), and the maximum length that could be dissected ranged from 4.7 cm to 33.9 cm (median, 17.0 cm; IQR 12.0–22.6 cm). The median diameter of the skin flap nourished by each perforator was 3.4 cm (range 0.1 cm—14.7 cm). Representative images are shown in Fig. 4.

### Image quality assessment

All the images were good for post-processing and analysis. The median (IQR) score of subjective image quality assessment was 4 (4–4.5). The agreement between two observers was good (ICC = 0.89, *p* < 0.001). The mean contrast-to-noise ratio was 25.3 ± 10.6.

### Surgical flap harvesting

**Fig. 5:**
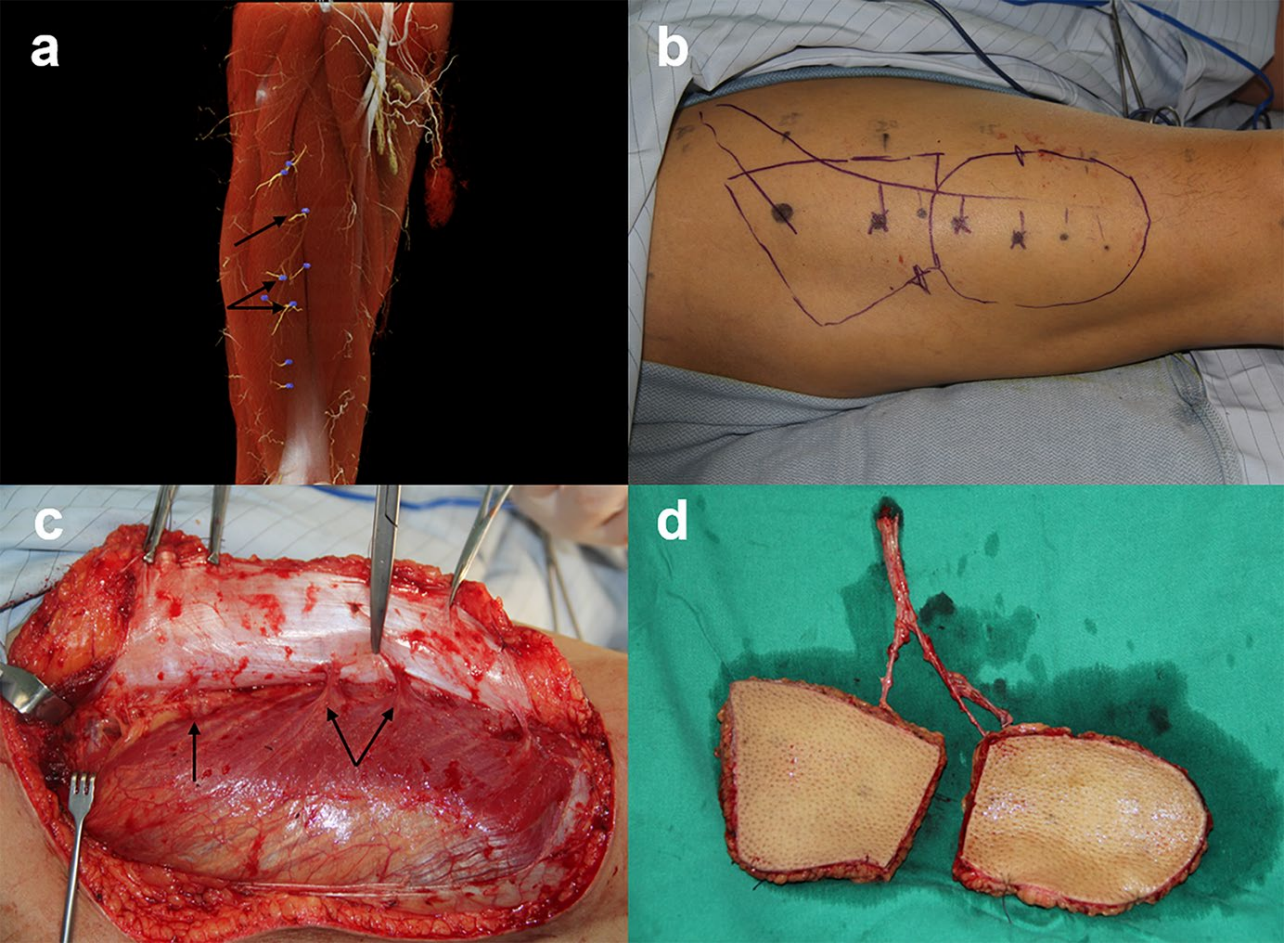
A case example of lobed flap harvesting guided by preoperative CTA mapping. **a–b** CTA mapping showed the location of perforators and guided the design of a lobed flap. **c–d** Perforators observed during the operation were consistent with the preoperative CTA assessment, and the lobed flap was successfully obtained

According to the condition of tumor resection or trauma repair, 28 patients underwent ALTP flap resection with the guidance of CTA. Two patients were conservatively treated, and 5 patients were lost to follow-up because they were not hospitalized for further treatment. Among the 28 patients with flap transplantation, 26 flaps survived and 2 patients had postoperative complications; the median follow-up time was 8.5 months (range 3–15 months). One case developed postoperative infection 2 days after surgery, and the other showed partial necrosis 5 days after surgery. Figure 5 shows a case example of lobed flap harvesting guided by preoperative CTA mapping.

## Discussion

The ALTP flap is the most popular choice for autologous soft tissue defect reconstruction because of its versatile design capability, increased pedicle length, and reduced donor site morbidity. The key to successful flap reconstruction is accurately predicting the location of ALTPs and determining their course through the skin. Many previous studies examined multiple imaging modalities [24–27], especially CTA, for preoperative evaluation of ALTP flap. Rao et al. [28] indicated that CTA was beneficial to the design and harvesting of individualized ALTP flap for the reconstruction of oral and maxillofacial soft tissue defects and achieved good functional and aesthetic outcomes. Schneider et al. [29] showed that preoperative CTA optimized the selection of flap donor site and reduced the operative time. In this study, we introduced a novel CTA technique to map the perforator anatomy of the flap to offer specific preoperative information (including origin, type, caliber, and length of the pedicle).

Appropriate CTA scan parameters and reconstruction models contribute to good imaging of the perforator. However, owing to limited imaging space, traditional CTA has great challenges in displaying peripheral perforator, even when the contrast agent is at its peak concentration [30]. In this study, we used the third-generation dual-source computed tomography with a modified scanning and post-processing procedure to improve the visibility of ALTPs. Compared to previous studies, more ALTPs have been found (5 perforators per thigh were found in this cohort). Hsieh et al. [6] reported an average of 3.1 perforators per thigh, while Chen et al. [31] and Cohen et al. [13] reported an average of 2.6 and 1.6 perforators per thigh, respectively. In addition, the distal secondary branch and its vascular territory were evaluated for the first time, which provided reliable preoperative guidance for flap design.

When performing the CTA scan, the following steps are required: Firstly, all patients must receive sublingual nitroglycerin before CTA acquisition. Nitroglycerin dilates small peripheral arteries and increases the number of assessable branches [32]. However, the use of dilators might affect the measurement of perforator size. The caliber measured on CTA should be taken as a preoperative guide, which could provide helpful information when the harvest of a dominant nutrient flap or a large skin paddle flap is considered. Secondly, based on the previous literature [5, 24, 33], the bolus-tracking monitoring section was set at the external iliac artery or CFA. Unfortunately, this high monitoring section results in a poor contrast agent concentration of distal perforators. Considering that most ALTPs originate from the descending branch of the LCFA, we adjusted the monitoring section to the middle segment of the descending branch to ensure good imaging. In fact, more perforators were identified in the distal zone, proving the scanning mode’s feasibility. Thirdly, a high convolution kernel can improve spatial resolution despite increases in image noise [34, 35]. However, as traditional CTA uses a medium soft tissue convolution reconstruction kernels, tiny distant vessels were omitted. The sharp convolution reconstruction kernel combined with iterative reconstruction can greatly reduce noise and ensure the visualization of perforators. Meanwhile, low tube voltage remarkably improves the enhancement degree of the perforator. As a result, the modified CTA scan protocol used in this study cohort had a good imaging efficacy for characterizing ALTPs, and the mean attenuation of the descending branch was 740.6 ± 118.5 HU.

Indeed, due to the great anatomic variation of ALTPs [19, 36], the identification of its anatomic origin and course is significant to surgeon’s selection of flap. A septocutaneous perforator facilitates harvesting, whereas musculocutaneous perforator is more suitable for musculocutaneous flap transfer. In this study, most ALTPs originated from the descending branch, which is consistent with the available literature [6, 13, 19, 31]; there was one patient with a high origin directly from the CFA identified in the present study. The high-origin perforator helped to obtain a longer vascular pedicle at harvest. Moreover, visualized images could better display the distribution and type of perforations. Compared with conventional volume rendering, CR uses different light maps to generate a realistic depiction of rendered data and provides a more photorealistic expression of tissue structures [37–39]. Most importantly, the new post-processing procedure allows the segmentation of different tissue structures. Through the identification and segmentation of perforators in maximum intensity projection images, perforators can be highlighted in CR images independent of the enhancement effect. Owing to the occlusion among tissues with different CT values, displaying anatomical details in a single CR image was difficult. Hence, perforators were divided into three anatomic levels for display, and the emerging location and projection direction were marked on different levels to provide “anchor points.”

Another important problem of CTA is accurately transferring the anatomic information to surgery guidance. Rozen et al. [40] first explored an intraoperative navigation technology in the location of perforators. However, this technology relies on a special navigator and adds to the risk of flap resection, and thus, it is an impractical procedure for surgeons. Furthermore, Shen et al. [5] introduced a virtual printed template projection technology for perforator localization, but the printed template used to mark perforators was not curved to the skin surface, resulting in inaccurate positioning. Therefore, a more convenient and precise method was presented in our study. The reticular position lines were drawn on patients’ thigh skin before scanning, and the location of perforators and the path of the main vessel were clearly mapped on the skin by vertical projection.

The present study has some limitations. First, this was a retrospective study with a small sample size, which may have restricted the description of anatomical variations. Second, the anatomical data of perforators observed during the operation were not analyzed and evaluated in detail. Yet, all the surgical flap harvests were successful with the guidance of preoperative CTA and most flaps (26/28) survived.

## Conclusion

This study shows that the proposed CTA mapping technique is a useful tool for the localization of ALTPs. With the guidance of preoperative CTA, surgeons could optimize flap design and harvesting.

## Data Availability

The datasets used and/or analyzed during the current study are available from the corresponding author on reasonable request.

## Abbreviations

ALTP: Anterolateral thigh perforator
CFA: Common femoral artery
CR: Cinematic rendering
CTA: Computed tomography angiography
IQR: Interquartile range
LCFA: Lateral circumflex femoral artery
ROI: Region of interest
